# Hypertension as a risk factor for atherosclerosis: Cardiovascular risk assessment

**DOI:** 10.3389/fcvm.2022.959285

**Published:** 2022-08-22

**Authors:** Anastasia V. Poznyak, Nikolay K. Sadykhov, Andrey G. Kartuesov, Evgeny E. Borisov, Alexandra A. Melnichenko, Andrey V. Grechko, Alexander N. Orekhov

**Affiliations:** ^1^Institute for Atherosclerosis Research, Moscow, Russia; ^2^Petrovsky National Research Centre of Surgery, Moscow, Russia; ^3^Laboratory of Angiopathology, Institute of General Pathology and Pathophysiology, Moscow, Russia; ^4^Federal Research and Clinical Center of Intensive Care Medicine and Rehabilitology, Moscow, Russia

**Keywords:** atherosclerosis, hypertension, cardiovascular disease, cardiovascular risk, vessels

## Abstract

Atherosclerosis is a predecessor of numerous cardiovascular diseases (CVD), which often lead to morbidity and mortality. Despite the knowledge of the pathogenesis of atherosclerosis, an essential gap in our understanding is the exact trigger mechanism. A wide range of risk factors have been discovered; however, a majority of them are too general to clarify the launching mechanism of atherogenesis. Some risk factors are permanent (age, gender, genetic heritage) and others can be modified [tobacco smoking, physical inactivity, poor nutrition, high blood pressure, type 2 diabetes (T2D), dyslipidemia, and obesity]. All of them have to be taken into account. In the scope of this review, our attention is focused on hypertension, which is considered the most widespread among all modifiable risk factors for atherosclerosis development. Moreover, high blood pressure is the most investigated risk factor. The purpose of this review is to summarize the data on hypertension as a risk factor for atherosclerosis development and the risk assessment.

## Atherosclerosis is the main cause of cardiovascular disease

Atherosclerosis is the main cause of cardiovascular diseases (CVD). Intima of middle- and large-sized arteries is most vulnerable to atherosclerosis, especially the sites of vessel branching. It can be explained by the nature of the blood flow since areas exposed to normal shear stress seem to be protected. One of the initial events in atherogenesis is the expression of adhesion molecules by activated endothelium. This allows mononuclear leukocytes, such as monocytes and T-cells, to attach to the endothelium and infiltrate the intima. Along with these cells, dendritic cells, mast cells, neutrophils, and B-cells may also be present in lesions. The essential type of cells present in the atherosclerotic lesion is the smooth muscle cells (SMCs). These cells change their phenotype to synthetic and migrate to the intima. The hallmark of atherosclerosis is the appearance of fatty streaks further evolving into atherosclerotic plaques. Atherosclerosis can induce CVD through stenosis and atherothrombosis, which are capable of decreasing blood flow. Atherothrombosis occurs when plaques are damaged by the effects of proinflammatory cytokines and chemokines on the fibrous cap. When plaques are damaged and ruptured, the prothrombotic material is exposed to the coagulation system, with the ensuing inhibition of blood flow and thus the induction of CVD (1).

For a very long time, CVD has been the leading global cause of premature mortality. According to statistics, by 2030, 23.6 million people will be dying from CVD every year. In northwestern and southern Europe, there is a moderate downward trend in mortality and morbidity due to CVD (2).

In Europe, CVD are the cause of 49% of deaths. It is the most important cause of premature mortality and Disability Adjusted Life Years (“DALYS”) in Europe, which attaches great importance to this topic in the field of public health. The annual cost of medical care for CVD in the European Union is about 192 billion euros (3). CVD can be caused by a large number of factors. Some of them are permanent (age, gender, genetic heritage) and others are changeable, that is, they can be influenced [tobacco smoking, physical inactivity, poor nutrition, high blood pressure, type 2 diabetes (T2d), dyslipidemia, obesity] (4). Risk factor control (45–75%) and proper treatment of CVD (25–55%) are responsible for reducing CVD mortality in highly developed countries (5).

## Cardiovascular risk factors

**FIGURE 1:**
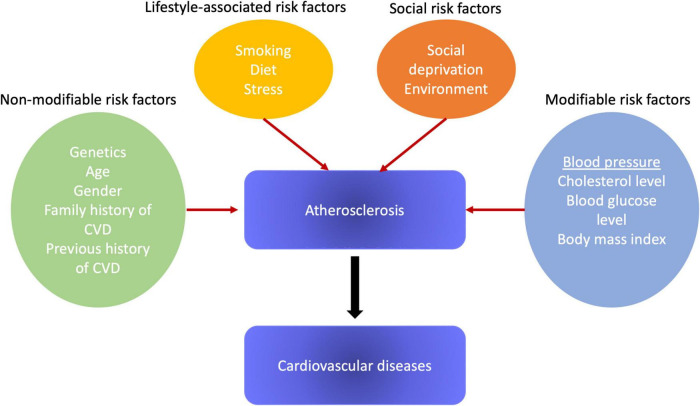
Risk factors for atherosclerosis development.

The main risk factors for atherosclerosis and, subsequently, CVD, are high blood pressure (BP), cigarette smoking, diabetes mellitus, and lipid metabolism disorders. Among them, high blood pressure is linked with the most convincing evidence of a causal relationship and has a high prevalence of exposure (6). In Figure 1 we provided the simplified scheme of various risk factors’ impact. However, there is concrete evidence that the biologically normal blood pressure level in humans is significantly lower than the level that is usually used both in clinical practice and research, which leads to an underrepresentation of the role of blood pressure as a risk factor for CVD (7). We put forward an integrated theory of the cause-effect relationship of CVD, which is confirmed by a reliable set of consistent evidence: CVD in humans are primarily caused by a right-sided shift in the distribution of blood pressure.

Due to the dominance of social networks, there are plenty of theories, but only a few satisfy the basic requirements of causality. Scientific theories are the most reliable because they are structured and can be refuted by systematic observation and experimental verification of the hypothesis testing. Our theory meets almost all the criteria of causality proposed by Bradford Hill. The changes that have arisen since the period of the post-industrial revolution have entailed consequences for the morbidity profile of the world’s population. As a result of technological progress, society is increasingly inclined to a sedentary lifestyle. This fact has led to an increase in the number of chronic diseases, such as obesity, T2D, and systemic hypertension, conditions known to be associated with increased cardiovascular risk (8).

As mortality and morbidity of CVD inexorably increased, the Framingham Study was launched to study risk factors and physiopathology associated with CVD. This prospective, long-term study made it possible to stratify cardiovascular risk as to the probability of a coronary event in the next 10 years. Since then, the Framingham score has been a practical method for assessing cardiovascular risk in various population groups (9).

This indicator makes it possible to assess the risk of coronary heart disease (CHD) for 10 years based on such parameters as blood pressure, systolic blood pressure (SBP), total cholesterol, HDL cholesterol, smoking, and antihypertensive treatment. Based on the calculated risk, an individual can be classified as having a low, medium, or high risk of developing CAD, including fatal coronary death or non-fatal myocardial infarction (MI) (10).

The risk factors for CAD include modifiable lifestyle factors, such as smoking, dyslipidemias, obesity, sedentariness, diabetes, and alcohol intake, as well as non-modifiable characteristics like age, sex, and family history. Among the modifiable risk factors, arterial hypertension is recognized as the key to ischemic diseases and disorders of cerebral circulation (11). A randomized study involved 3,845 participants with an average age of 83 years; this study revealed that lowering BP from 161/84 to 144/78 mmHg reduces the risk of cerebral circulatory disorders by 30% and cardiovascular events by 23% (12).

### Smoking

Another significant modifiable risk factor is smoking. It is known that smokers over the age of 60 have doubled risk of atherosclerosis and subsequent CVD compared to non-smokers. For people under the age of 60, the risk is five times higher (4). In addition to CV risk, smoking is linked with a higher prevalence of chronic kidney disease (CKD). In an observational study of 65,589 people who were observed for 10.3 years, it was shown that the risk of developing CKD is 4 and 3.3 times higher in current and former smokers, respectively, compared with non-smokers (13).

### Diet-associated risk factors

Controlling lifestyle-related factors, including diet and exercise, is fundamental to preventing atherosclerosis. Atherogenic and hypocaloric diets lead to hypertension, diabetes, dyslipidemia, overweight, and other disorders. Compared with those patients who do not suffer from diabetes, patients with diabetes have hypertension twice as often (14). Diabetes is also one of the most crucial risk factors for determining CAD, so the presence of this pathology is considered a risk factor equivalent to a heart attack, that is, despite the absence of any CV signs, diabetics are classified as “high cardiovascular risk” (15).

The Framingham study reports that high triglyceride levels and low HDL cholesterol lead to an elevated risk of CV. Based on data from the same Framingham study, it was found that obesity is the cause of 26% of cases of hypertension in men and 28% in women, and approximately 23% of cases of CHD in men and 15% in women (16).

However, it should be noted that the traditional Framingham indicator was developed in the 1950s and confirmed in the 1960s and 1970s when the prevalence of overweight and obesity in the United States was 1/3 of the current figure and acute MI was more common among men (17). Since the 1980s, heart attacks have become increasingly common among women, and obesity has become a key global problem. Therefore, this indicator may underestimate the CV risk in the modern population (18).

Thus, to elevate the positive prognostic value of CAD in the traditional Framingham risk scale, it was proposed to add factors that suggest subclinical atherosclerotic disease, called emerging factors, which include peripheral vascular diseases, thickening of the intima-media wall of the carotid artery, and calcium content in the coronary arteries, which contribute to an elevation in CV levels since they are markers of endothelial damage (19).

### Combination of risk factors

Moreover, to assess cardiovascular risk, other factors, such as C-reactive protein and the presence of metabolic syndrome, and traditional risk factors, such as a family history of premature CHD, should be taken into account (20). Also, other risk factors, namely aggravating factors, were also added to the traditional Framingham scale: left ventricular hypertrophy (LVH) *via* electrocardiography, microalbuminuria (30–300 mg/24 h), and CKD (plasma creatinine levels higher than 1.5 mg/dL or creatinine filtration below 60 mL/min) (21). According to this, the presence of one of these factors elevates the risk score to a higher level in contrast to the results that were obtained as a result of applying the traditional Framingham score.

In this regard, there is a question of whether the assessment of CV risk in patients with arterial hypertension will increase the probability of coronary events for 10 years by taking into account aggravating risk factors. Since CVD entails social and economic losses, the study of the alleged coronary risks gives the prospect of applying more appropriate therapeutic measures, thus preventing these events (22). The objective of this study was to assess cardiovascular risk in patients with arterial hypertension using the traditional Framingham risk assessment in comparison with the assessment modified by the inclusion of new risk factors.

## Hypertension

In the course of epidemiological studies, arterial hypertension was found to be the most significant modifiable CV risk factor, accounting for 48% of all strokes and 18% of all coronary events. Thus, antihypertensive treatment is still the cornerstone of primary and secondary prevention of CVD. Despite these statistics, only about 40% of patients with arterial hypertension receive medication (antihypertensive agents) and only 1/3 of them are successfully treated for normotension (23). To analyze the effect of antihypertensive drugs on cardiovascular risk, various research schemes were used, the outcomes of which were ambiguous. Population studies have revealed a positive association of antihypertensive agents with coronary artery calcification (CAC) and the progression of CAC. Associations with clinical CV events were less consistent; some studies demonstrated a markedly elevated risk, while in others the risk when taking antihypertensive drugs was not noticed (24). The results of randomized placebo-controlled trials (RCTs) were also ambiguous and depended on the specific outcome under consideration and the characteristics of the patient: gender, race, and the presence of additional CV risk factors (25).

When analyzing the relationship between antihypertensive and cardiovascular risk, it is extremely important to determine whether blood pressure is effectively controlled. Several RCTs have demonstrated that a more active, compared with a less active decrease in blood pressure, leads to a lower risk of stroke and CV events; however, this does not apply to coronary events (26, 27). In the REGARDS (Reasons for Geographic and Racial Differences in Stroke) trial (28), participants with blood pressure lowered to the ideal level still had a higher risk of stroke in contrast to those participants who reached the ideal blood pressure level without resorting to treatment. The same was also observed in Multi-Ethnic Study of Atherosclerosis (MESA) trial (29) for coronary and cardiovascular events. The key difficulty with such drug epidemiology is the indication since the distribution of treatment in epidemiological studies is not randomized, and indications for antihypertensive treatment may be linked with the risk of CVD.

## Hypertension in atherosclerosis

Hypertension is likely to affect the arterial tree by thickening artery walls, the development of atherosclerotic plaques, and their vulnerability to rupture. However, there are almost no data on the difference in the impact of a wide range of BP values (normal, high-normal, pre-hypertension, and overt hypertension) on atherosclerosis development. Since 2017, the American Heart Association/American College of Cardiology (AHA/ACC) (30, 31) and the European Society of Cardiology (ESC) (32) have diverged in the definition of hypertension. ESC maintained the previous definition (SBP 140–149 mmHg, DBP 90–99 mmHg) (32) and the AHA/ACC adopted a lower threshold to define hypertension (SBP 130–139 mmHg, DBP 80–89 mmHg) (30). Despite these criteria being inconsistent, both guidelines give advice to control elevated BP by non-pharmacological interventions in the first instance and to start treating with antihypertensive drugs only in case the risk becomes high. The association between BP and cardiovascular events is linear (33), cut-offs are used to categorize BP as optimal, normal, high-normal, or hypertension. Recent studies revealed that the risk associated with high-normal BP is also in charge. Thus, Whelton et al. demonstrated a positive association of a rise in BP with coronary artery calcium prevalence, as well as the incidence of atherosclerosis-linked cardiovascular events using data from the MESA. In those who do not have classical risk factors and whose systolic BP (SBP) < 130 mmHg, the occurrence of atherosclerotic lesions, as well as the risk of incident adverse events increase in step with SBP increases above 90 mmHg (34–36).

The Framingham Heart study demonstrated that high normal blood pressure and hypertension (stage 1, stage 2, and higher) elevate the risk of CHD in both men and women (37). The Japanese urban cohort study (Suita Study) revealed that high normal BP and hypertension of the 1st and 2nd stages or higher lead to an increased risk of MI in men, and that hypertension of stage 2 or higher increases the risk of MI in women (38). We have summarized the data on the effects of blood pressure elevation on cardiovascular outcomes in Table 1.

**TABLE 1:** Effects of various rates of hypertension on cardiovascular outcomes.

Study	Subjects	Hypertension	Effect	References
Framingham heart study	Women	High normal blood pressure	Increased risk of CHD	Garcia et al. (37)
Framingham heart study	Women	Hypertension stage 1	Increased risk of CHD	Garcia et al. (37)
Framingham heart study	Women	Hypertension stage 2	Increased risk of CHD	Garcia et al. (37)
Framingham heart study	Men	High normal blood pressure	Increased risk of CHD	Garcia et al. (37)
Framingham heart study	Men	Hypertension stage 1	Increased risk of CHD	Garcia et al. (37)
Framingham heart study	Men	Hypertension stage 2	Increased risk of CHD	Garcia et al. (37)
Japanese urban cohort study (Suita study)	Men	High normal blood pressure	Increased risk of MI	Park (38)
Japanese urban cohort study (Suita study)	Men	Hypertension stage 1	Increased risk of MI	Park (38)
Japanese urban cohort study (Suita study)	Men	Hypertension stage 2	Increased risk of MI	Park (38)
Japanese urban cohort study (Suita study)	Women	Hypertension stage 2	Increased risk of MI	Park (38)
Meta-analysis by the Japan arteriosclerosis longitudinal study group	Men	Higher SBP, pulse pressure and average blood pressure	Increased risk of MI (1.2 per 1,000 person-years)	Hussain et al. (41), Sobenin et al. (42), Andersson et al. (43)
Meta-analysis by the Japan arteriosclerosis longitudinal study group	Women	Higher SBP, pulse pressure and average blood pressure	Not increased risk of MI (0.5 per 1,000 person-years)	Hussain et al. (41), Sobenin et al. (42), Andersson et al. (43)

Meta-analysis of individual data for 1 million adults examined in 61 prospective studies revealed that over the entire range of values of normal SBP decreasing to 115 mmHg, the slope of the relationship between mortality from CHD (plotted on a double scale) and normal levels of SBP was approximately constant in each age range, although the relative strength of the association was weaker for CHD than for stroke mortality in middle ages (39). Moreover, for the relationship between mortality from CHD and the usual values of diastolic blood pressure (DBP) decreasing to 75 mmHg, the age-related HRs associated with differences of 10 mmHg in the usual DBP are equivalent to those associated with differences of 20 mmHg in the usual values of SBP (40).

The Japan Arteriosclerosis Longitudinal Study Group conducted a meta-analysis of 16 cohort studies, numbering 48,224 Japanese men and women (40–89 years old) at the initial stage and an average of 8.4 years of follow-up (41, 42). A higher SBP, pulse pressure, and average blood pressure led to an increase in the risk of MI in men, but not in women. The incidence of MI was 1.2 and 0.5 per 1,000 person-years in men and women, respectively. Due to the small sample size of women, the relationship between higher blood pressure values and incident MI may not be observed in women (43).

A recent study by Gonzalez-Guerra et al. showed that in the preclinical model of atherosclerosis, the mechanical effect of mild BP increase directly stimulates the progression of atherosclerotic lesions independent of the RAAS pathway activation. This finding is consistent with the hypothesis that not only hypertension, but even non-optimal BP is a risk factor for the progress of atherosclerosis (44).

Several compound classes appeared to have antihypertensive effect. Among such drugs, there are ACE (angiotensin-converting enzyme) inhibitors, calcium-channel blockers, ARB (angiotensin receptor blockers) inhibitors, beta-adrenergic blockers, diuretics (thiazides/thiazide-like diuretics/loop diuretics/potassium-sparing diuretics), vasodilators (hydralazine/minoxidil), and others. However, the use of antihypertensive drugs was sown to improve only blood pressure, but not the CVD risk (45).

## Hypertension and total cardiovascular risk

The concept is based on the fact that only a small part of the population with hypertension has an isolated increase in BP, while the majority have additional cardiovascular risk factors. In addition, the combination of BP and other risk factors can reinforce each other, which results in a total cardiovascular risk that exceeds the sum of its individual components (46). After all, in high-risk individuals, antihypertensive treatment strategies, such as initiation and intensity of treatment, the use of drug combinations, etc., as well as other treatment methods, may differ from those used in lower-risk individuals (47).

In people at high risk, blood pressure control is difficult and requires a more frequent combination of antihypertensive drugs together with other therapy, primarily statin treatment. The therapeutic approach should consider the total CV risk in addition to BP levels to increase the cost-effectiveness of hypertension treatment (48).

## Assessment of total cardiovascular risk

It is not difficult to assess the total cardiovascular risk in certain subgroups of patients, for example, in patients with previous CVD, diabetes, CHD, or with highly elevated single risk factors. With all these conditions, the total CV risk is high or extremely high, so there is a need for intensive treatment, which reduces the CV risk (49). Nevertheless, quite a lot of patients with arterial hypertension do not belong to any of these categories, and the identification of patients with low, moderate, high, or very high risk requires the use of models to assess the total cardiovascular risk, which will allow the therapeutic approach to be properly adjusted (50).

To assess the total cardiovascular risk, several computerized methods have been developed. Their meanings and limitations were also considered. The SCORE model was developed based on large European cohort studies. This model evaluates the risk of death from CV and not only coronary diseases for 10 years, depending on age, gender, smoking habits, total cholesterol, and SBP (51, 52). The SCORE model makes it possible to calibrate graphs for individual countries, which has been done for many European countries. At the international level, two sets of charts are set: one for high-risk countries and one for low-risk countries (53). An electronic interactive version of SCORE---Heart Score^1^ also takes into account the effect of LDL cholesterol on total CV risk. The charts and their electronic versions can help in risk assessment and management, but must be interpreted in the light of the clinician’s knowledge and experience, especially about local conditions. In addition, the conclusion that the assessment of total cardiovascular risk is associated with improved clinical outcomes compared to other strategies was not sufficiently studied in a randomized trial (54, 55).

The cardiovascular risk may be higher than indicated in the tables in people who have a sedentary lifestyle and in people with central obesity; the high relative risk linked with being overweight is higher in young people than in older people. Socially disadvantaged people and members of certain ethnic minorities may also have a higher risk of CVDs. Individuals with elevated fasting glucose levels and/or an abnormal glucose tolerance test who do not meet the diagnostic criteria for T2D belong to the same category. The same applies to patients with elevated levels of triglycerides, fibrinogen, apolipoprotein B, lipoprotein A, and highly sensitive C-reactive protein. Also, people with a family history of premature CVD under the age of about 60 years may have a high CV risk (56).

In the SCORE model, the total CV risk is expressed as the absolute risk of death from CVD within 10 years. Due to the strong age dependence in young patients, the absolute total CV risk may be low even in the presence of high BP with additional risk factors (57). But with insufficient treatment, this condition can result in an irreversible high-risk condition years later. In younger patients, when choosing a treatment, it is better to follow a quantitative assessment of relative risk or an assessment of the age of the heart. The table of relative risk is available in the Joint European Societies’ Guidelines for the prevention of CVD in clinical practice, which is convenient when consulting young people (58, 59).

Additional attention was paid to the detection of asymptomatic organ damage since asymptomatic changes in several organs associated with hypertension indicate a steady development in the continuum of CVD, which significantly increases the risk exceeding the risk caused by the mere presence of risk factors. Thus, the search for asymptomatic organ damage can be significant whenever evidence of additional risk is discussed (60).

Additional emphasis was placed on the detection of asymptomatic organ damage since hypertension-related asymptomatic changes in the continuum of CVD greatly elevate the risk exceeding the risk caused by the mere presence of risk factors (50).

International guidelines for the management of hypertension like the 1999 and 2003 World Health Organization/International Society of Hypertension guidelines, 2003, 2007, and 2013 European Society of Hypertension/European Society of Cardiology guidelines, and the 2012 European Society of Cardiology prevention guidelines have stratified CV risk in different categories based on BP category, CV risk factors, asymptomatic organ damage, and the presence of diabetes or symptomatic CVD or CKD (61). The classification by low, moderate, high, and very high risk refers to a 10-year increase in mortality from CVD, as defined in the prevention guidelines of the European Society of Cardiology 2012.

## Conclusion

Numerous investigations proclaim the association between increased blood pressure and atherosclerosis. The results of these studies are reflected in guidelines and risk assessment scores. However, there is still no definitive data on the effects of various levels of increased BP (mild increase, severe increase, etc.), as well as the causal relationship. Of course, all these factors affect the efficiency of models for assessing cardiovascular risk and thus alter the detection and prevention success (62, 63).

To date, all existing models for assessing cardiovascular risk have limitations that need to be taken into account. The importance of damage to target organs in determining the total risk depends on how scrupulously the damage is assessed based on available facilities. For example, the rationale for assessing total CV risk is to make optimal use of limited resources for the prevention of atherosclerosis and CVD or to evaluate preventive measures in accordance with the elevated risk (64). However, absolute risk stratification is commonly used by private or public health care providers to determine the barrier below which treatment is not recommended. Any threshold for determining a high total CV risk is arbitrary, as is the use of limit values resulting in intensive interventions above this threshold and no actions below. Hypertension is one of the strongest risk factors for almost atherosclerosis, subsequent CVDs, as well as for cardiac events. The difference between high normal BP and hypertension is based on arbitrary limit values, and hypertension is the level at which intervention to lower BP has preventive benefits, which is confirmed by a number of documents. Atherosclerosis prevention and guidelines for the treatment of moderately elevated BP should be associated with a quantitative assessment of the total CV risk (65–67).

## Author contributions

AP: writing—original draft preparation. NS, AK, EB, AM, AG, and AO: writing—review and editing. All authors contributed to the article and approved the submitted version.

## Footnotes

1
www.heartscore.org
